# Supplementary material for: The value of artificial intelligence in the diagnosis of lung cancer: A systematic review and meta-analysis
Source: PLoS One. 2023 Mar 23;18(3):e0273445. doi: 10.1371/journal.pone.0273445 (PMC10035910; doi:10.1371/journal.pone.0273445)
Supplement: S1 Data — (DOCX) [file pone.0273445.s002.docx]

| Inclusion in the study | Year of publication | AI algorithms | Total sample size | TP | FP | FN | TN |
| --- | --- | --- | --- | --- | --- | --- | --- |
| Sun | 2013 | Support vector machines | 33 | 15 | 2 | 2 | 14 |
| Teramoto | 2019 | Random Forest | 43 | 24 | 13 | 1 | 5 |
| Wang | 2016 | Support vector machines | 193 | 91 | 15 | 31 | 56 |
| Yin-Chen Hsu | 2020 | Artificial Neural Network ANN | 234 | 6 | 34 | 2 | 192 |
| Li Tian | 2020 | Computer-aided diagnosis | 109 | 65 | 10 | 30 | 4 |
| Xu Liping | 2014 | fuzzy neural network | 44 | 19 | 2 | 2 | 21 |
| Dilger | 2015 | Artificial neural network | 50 | 20 | 2 | 2 | 26 |
| Dilger | 2015 | Linear discriminant analysis | 50 | 17 | 3 | 5 | 25 |
| Manikandan | 2016 | Support vector machine | 257 | 22 | 16 | 0 | 219 |
| Silva | 2017 | Convolutional neural network | 200 | 98 | 9 | 2 | 91 |
| Li | 2018 | Random forest1 | 100 | 17 | 13 | 3 | 63 |
| Li | 2018 | Random forest2 | 200 | 62 | 22 | 8 | 108 |
| Li | 2018 | Random forest3 | 300 | 52 | 22 | 6 | 220 |
| Li | 2018 | Random forest4 | 400 | 120 | 16 | 16 | 248 |
| Li | 2018 | Random forest5 | 500 | 147 | 31 | 13 | 309 |
| Li | 2018 | Random forest6 | 600 | 184 | 40 | 16 | 360 |
| Ren | 2019 | Manifold regularized classification deep neural network | 245 | 70 | 8 | 16 | 151 |
| Ren | 2019 | Classification deep neural network | 245 | 54 | 10 | 32 | 149 |
| Dilger | 2013 | Artificial neural network | 27 | 10 | 2 | 0 | 15 |
| Duan | 2020 | Artificial neural network1 | 204 | 84 | 14 | 44 | 62 |
| Duan | 2020 | Support vector machine1 | 204 | 88 | 31 | 40 | 45 |
| Duan | 2020 | Artificial neural network2 | 78 | 43 | 5 | 3 | 27 |
| Duan | 2020 | Support vector machine2 | 78 | 40 | 7 | 6 | 25 |
| Duan | 2020 | Artificial neural network3 | 33 | 15 | 2 | 1 | 15 |
| Duan | 2020 | Support vector machine3 | 33 | 14 | 3 | 2 | 14 |
| Chamberlin | 2021 | Artificial neural network ANN | 117 | 69 | 0 | 14 | 34 |
