# Supplementary material for: The value of artificial intelligence in the diagnosis of lung cancer: A systematic review and meta-analysis
Source: PLoS One. 2023 Mar 23;18(3):e0273445. doi: 10.1371/journal.pone.0273445 (PMC10035910; doi:10.1371/journal.pone.0273445)
Supplement: Attachment — Submitted filename: Response to Reviewers.docx [file pone.0273445.s005.docx]

Dear reviewer:

Thank you for your decision and constructive comments on my manuscript. We have carefully considered the suggestion of Reviewer and make some changes. We have tried our best to improve and made some changes in the manuscript.

The red part that has been revised according to your comments. Revision notes, point-to-point, are given as follows:

- Journal Requirements:

1.Please review your reference list to ensure that it is complete and correct. If you have cited papers that have been retracted, please include the rationale for doing so in the manuscript text, or remove these references and replace them with relevant current references. Any changes to the reference list should be mentioned in the rebuttal letter that accompanies your revised manuscript. If you need to cite a retracted article, indicate the article’s retracted status in the References list and also include a citation and full reference for the retraction notice.

We have reviewed and made the necessary changes to the reference list as per your request. The reference list is now complete and correct. We have added or replaced new references to studies numbered 41, 43, 24, 22, 10, and 33 to 39 in the new manuscript, and have removed studies numbered 2,30, 32 to 34, 36 to 41, and 43 to 47 in the old manuscript.

- Response to Reviewer #2

1-Some of the symbols are not clear to the reader what they stand for. Therefore, a table of all symbols used in the paper will enhance the readability of the paper. It would be best if you had a separate table for all symbols in the article.

Thank you for your valuable feedback. We have added Table 5 on page 25 in the new manuscript. This table is used to explain the symbols that appear in the text.

2- Review the English to correct some grammar mistakes.

Thank you for your suggestions. We have reviewed the manuscript and corrected any grammatical errors.

- Response to Reviewer #3

1-Based on the final results of the query, authors ended up with 21 papers, 16 are written in Chinese and 5 in English. In the world of publication/research, English is considered the ‘official’ language - for many reasons. Some interested readers wouldn’t be able to refer to those 16 papers for further reading. Consequently, one of the critical criteria of the query in such a manuscript should be the language of the manuscript to be set to English.

Thank you for your suggestions. After careful consideration, we have kept a portion of the valuable Chinese literature from the original 21 references and incorporated new English references. As a result, a total of 14 references have been included in the research, with 12 being English references and 2 being Chinese references.

Sincerely,

Ke Tao

College of Life Science , Sichuan University, Chengdu610041, China.

Email: taoke@scu.edu.cn
